# Validation of tumour models for use in anticancer nanomedicine evaluation: the EPR effect and cathepsin B-mediated drug release rate

**DOI:** 10.1007/s00280-013-2209-7

**Published:** 2013-06-25

**Authors:** Ruth Duncan, Yee-Nee Sat-Klopsch, Angelika M. Burger, Michael C. Bibby, Heinz H. Fiebig, Edward A. Sausville

**Affiliations:** 1 Centre for Polymer Therapeutics, The School of Pharmacy, 29-39 Brunswick Square, London, WC1N 1AX UK; 2 Oncotest GmbH, Institute for Experimental Oncology, Am Flughafen 12-14, 79108 Freiburg, Germany; 3 Institute of Cancer Therapeutics, University of Bradford, Richmond Rd, Bradford, West Yorkshire BD7 1DP UK; 4 Marlene and Stewart Greenebaum Cancer Center, University of Maryland, 22 S. Greene Street, Room S9D07, Baltimore, MD 21201 USA; 5 Present Address: c/o Polymer Therapeutics Laboratory, Centro de Investigacion Príncipe Felipe, Av. Autopista del Saler 16 E, 46012 Valencia, Spain

**Keywords:** EPR effect, Cathepsin B, Evans Blue, HPMA copolymer–doxorubicin, Nanomedicines

## Abstract

**Purpose:**

Intravenously (i.v.) administered nanomedicines have the potential for tumour targeting due to the enhanced permeability and retention (EPR) effect, but in vivo tumour models are rarely calibrated with respect to functional vascular permeability and/or mechanisms controlling intratumoural drug release. Here the effect of tumour type and tumour size on EPR-mediated tumour localisation and cathepsin B-mediated drug release was studied.

**Methods:**

Evans Blue (10 mg/kg) and an *N*-(2-hydroxypropyl)methacrylamide (HPMA) copolymer–doxorubicin (Dox) conjugate (FCE28068) (5 mg/kg Dox-equiv) were used as probes and tumour levels (and Dox release) measured at 1 h after i.v. administration in a panel of murine and human xenograft tumours.

**Results:**

Evans Blue and FCE28068 displayed similar tumour levels in the range of 2–18 % dose/g at 1 h for B16F10 and L1210. Approximately half of the tumour models evaluated exhibited tumour size-dependent accumulation of FCE28068; smaller tumours had the highest accumulation. Administration of free Dox (5 mg/kg) produced tumour levels of <2.5 % dose/g independent of tumour size. Whereas the degree of EPR-mediated targeting showed ~12-fold difference across the tumour models evaluated, Dox release from FCE28068 at 1 h displayed ~200-fold variation.

**Conclusions:**

Marked heterogeneity was seen in terms of EPR effect and Dox release rate, underlining the need to carefully calibrate tumour models used to benchmark nanomedicines against known relevant standard agents and for optimal development of strategies for late pre-clinical and clinical development.

**Electronic supplementary material:**

The online version of this article (doi:10.1007/s00280-013-2209-7) contains supplementary material, which is available to authorized users.

## Introduction

An increasing number of nanomedicines, including liposomes, polymer conjugates, block copolymer micelles, nanoparticles and other complex hybrid technologies, are being developed as anticancer therapeutics, imaging agents and theranostics (reviewed in [1–4]). Several products are already in routine clinical use (e.g. Doxil^®^, Abraxane^®^) with a growing number of technologies including polymer therapeutics in clinical development [2, 3]. Intravenously (i.v.) administered long-circulating nanosized constructs have long been known to exhibit passive tumour targeting due to the enhanced permeability and retention (EPR) effect [5, 6]. This phenomenon has been demonstrated in many in vivo tumour models and the features of polymer conjugates [7, 8], nanoparticles [4] and block copolymer micelles [9] governing extravasation, as well as tumour characteristics [10] regulating the efficiency of the process have been discussed. Liposomal and polymer conjugate-based gamma camera imaging probes have also demonstrated the EPR effect in some tumours in patients [11–13]. A diverse array of tumour pathophysiological features regulate the efficiency of EPR-mediated tumour targeting including heterogeneity of intratumoural blood flow, angiogenic vascular permeability, tumour microenvironment including extracellular matrix features, interstitial pressure and lymphatic drainage, and thus, no single biomarker adequately predicts tumour targeting and/or antitumour activity [14].

**Fig. 1:**
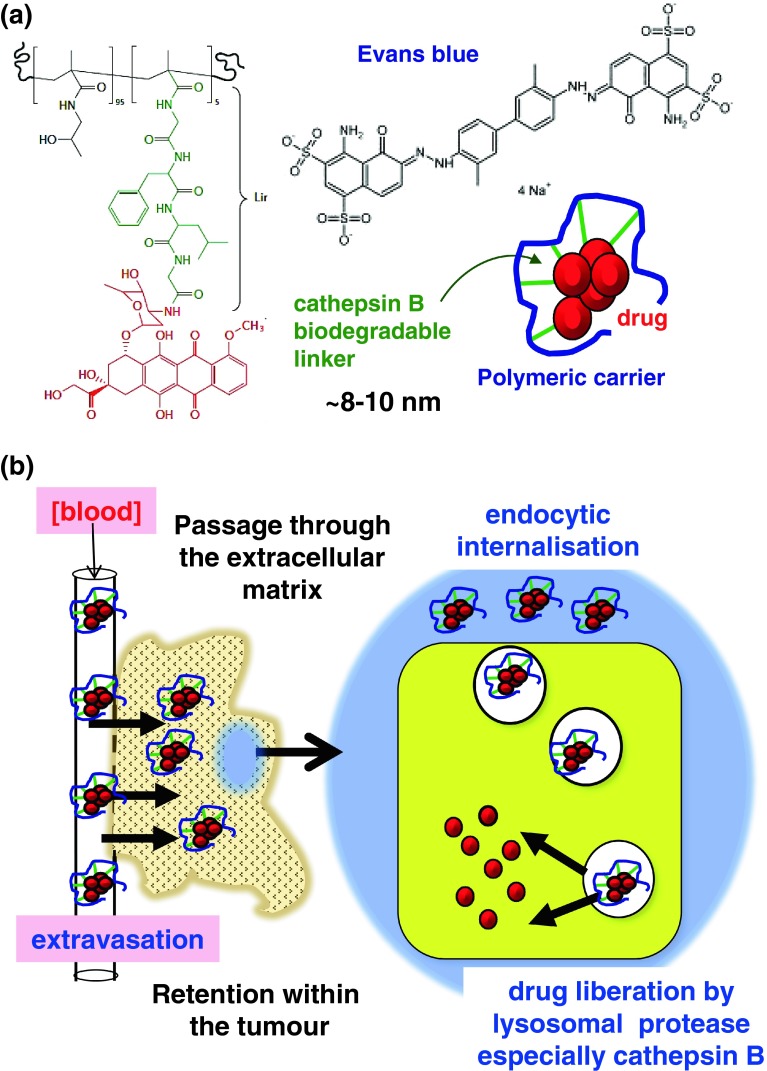
Structure of **a** FCE28068, Evans Blue and a schematic representation of pendant Dox molecules conjugated to a nanomedicine and **b** a schematic diagram showing the key steps in tumour targeting and lysosomotropic activation

As a functional EPR effect is the primary factor governing nanomedicine tumour access (Fig. 1), it is surprising that tumour models used to evaluate pharmacokinetics (PK) and/or antitumour activity have been rarely pre-calibrated for this characteristic. This makes comparison of results, both between experiments and between laboratories, difficult and prevents effective benchmarking of emerging technologies against those for which there is now a considerable clinical database. Moreover, although many constructs have been designed for intratumoural activation, particularly intralysosomal drug release (using low pH or lysosomal enzymes such as cathepsin B) (Fig. 1), few tumour models are calibrated with respect to these activation mechanisms. Therefore, the aim of this study was to quantify systematically the early-phase EPR-mediated tumour targeting using a panel of murine and human xenograft tumours. The albumin-binding dye Evans Blue, widely used as a physiological marker of vascular permeability [5], and *N*-(2-hydroxypropyl)methacrylamide (HPMA) copolymer–GFLG–Dox (FCE28068) (Fig. 1), a conjugate that has undergone Phase I/II clinical trials [11, 12], were selected as probes of the EPR effect in tumour models. In certain experiments, doxorubicin (Dox) alone was used as a reference control. To establish the comparability of Evans Blue and FCE28068 as EPR probes, experiments were initially conducted in a subcutaneous (s.c.) B16F10 model previously used widely to document the PK and antitumour activity of polymer conjugates [15] and a s.c. L1210 model known to be sensitive to Dox. FCE28068 was then used to quantify the effect of tumour size on early-phase (1 h) tumour accumulation in a panel of murine and human xenograft tumours (Table 1) and to study the effect of tumour size on passive targeting. In addition, as cathepsin B mediates Dox liberation from FCE28068, and this enzyme is also responsible for activation of polyglutamic acid anticancer conjugates currently in clinical trials (e.g. paclitaxel poliglumex (PPX) [16]), the extent of Dox released at 1 h was also quantified. It should be noted that the 1 h time point was selected for this study as it minimises additional kinetic effects introduced by differences in blood clearance rate, probe- or tumour model-dependent tumour efflux rates and/or inter-tumoural differences in the degradation rate of biodegradable probes, e.g. proteolysis of albumin over time [8, 17].

**Table 1:** Summary of the tumour models used

Code	Type of tumour	Mouse
*Murine models*		
L1210	Lymphocytic leukaemia	DBA2
B16F10^a^	Melanoma	C57 Blk
MAC 15A^b^	Adenocarcinoma	NMRI
MAC 26^b^	Adenocarcinoma	NMRI
Meta 7	Lung tumour	Balb/c
*Human xenografts*		
RXF 1220	Renal cell carcinoma	nu/nu
RXF 486	Renal cell carcinoma	nu/nu
PAXF 546	Pancreatic carcinoma	nu/nu
MEXF 276	Melanoma	nu/nu
MAXF 449	Mammary carcinoma	nu/nu
COR L23	Human non-small cell lung carcinoma	nu/nu
SK-N-SH	Neuroblastoma	SCID
IMR 32	Neuroblastoma	SCID
SK-N-DZ	Neuroblastoma	SCID

^a^[15]

^b^[14] and for others [17, 29]

## Materials and methods

### Materials and cells

HPMA copolymer–GFLG–Dox (FCE28068; also known as PK1) (MW ~ 30,000 g/mol; MW/Mn = 1.3; total Dox 6–8 wt%; free Dox < 1 % in respect of total) and Dox were a gift from Pharmacia and Upjohn, Italy. Evans Blue was purchased from Sigma, UK. The tumour models used are summarised in Table 1. B16F10 cells were donated by Prof. Ian Hart, St. Thomas’s Hospital, London, UK. L1210 cells were from Imperial Laboratories Ltd., UK. Meta-7 and COR L23 cells were from European Collection of Cell Cultures (ECACC), Wiltshire, UK. C57 black, DBA_2_, Balb-c and nu/nu mice were supplied by Bantin and Kingman Ltd., UK. All animal experiments were performed in accordance with the United Kingdom Co-ordinating Committee on Cancer Research (UKCCCR) Guidelines for the Welfare of Animals in Experimental Neoplasia (1998) [17] and with UK Home Office Guidelines.

### Tumour models and administration of probes

All tumours were established as s.c. models (see Table 1 and [14, 15, 17] for full details). Generally, cells were implanted into the posterior right flank of mice, and the studies were initiated when tumour reached 25–289 mm^2^ (product of two diagonal width). MAC 26 tumours were implanted as tumour pieces [14]. Evans Blue (2 mg/mL in 0.9 % NaCl; 10 mg/kg), FCE28068 or free Dox (1 mg/mL in PBS; 5 mg/kg Dox-equiv) was administered i.v. via the tail vein. FCE28068 was given the higher dose of 40 mg/kg Dox-equiv to NMRI mice bearing the murine adenocarcinomas MAC 15A and MAC 26 to enable comparison with efficacy experiments in this model [14]. After 1 h, all animals were humanely killed, and tumour was carefully removed, washed with PBS, weighed and snap-frozen in liquid nitrogen before extraction and analysis. It should be noted that most of this study was conducted according to the 2nd Edition of the UKCCCR Guidelines for the Welfare of Animals in Experimental Neoplasia [18], which states that the maximum tumour burden should not exceed 10 % of the host animal’s normal body weight or 2.5 g. The initial study involving measurement of Evans Blue levels in the s.c. L1210 tumour model was conducted prior to the 2nd Edition of UKCCCR Guidelines, thus accounting the use of tumours in excess of 2.5 g.

### Quantitation of Evans Blue in tumour samples

Evans Blue was extracted from the tumour samples using a method adapted from Harada et al. (1971) [19]. Briefly, the tissue samples were blade homogenised in 2 mL of PBS. The homogenate (1,900 μL) was mixed with aqueous sodium sulphate solution (0.5 % w/v, 3 mL) and acetone (7 mL) in polypropylene tubes and then incubated at room temperature (20 °C) overnight before centrifugation (1,000×*g*, 30 min, 20 °C) to precipitate the tissue. The concentration of Evans Blue in the supernatant was quantified spectrophotometrically at 590 nm using a standard curve prepared by spiking foetal bovine serum (FBS) samples with known concentrations of Evans Blue.

### Quantitation of free Dox in tumour samples

Following i.v. administration of free Dox, drug in tumour samples was quantified using a previously described HPLC method [20, 21]. Throughout the extraction procedure, all samples were maintained at 4 °C and kept in the dark (wrapped in foil). Polypropylene tubes and HPLC inserts were used to minimise adsorption of Dox to containers. Briefly, tumour samples were thawed, homogenates were prepared in PBS, and samples (900 μL) were placed in polypropylene tubes. A daunomycin (Dnm) standard (donated by Rhône-Poulenc, France) (100 ng, 100 μL) in PBS was added as an internal standard, followed by ammonium formate buffer (1 M; pH 8.5; 100 μL) and chloroform/propan-2-ol (4:1 v/v, 5 mL). After thorough vortex-mixing (3 × 10 s; within 30 min), the tubes were centrifuged (1,000×*g*; 30 min, 10 °C). The upper aqueous layer and tissue pellet were then carefully removed. The remaining organic phase was evaporated to dryness using a sample concentrator under constant nitrogen gas (N_2_) flow over 20 min. The samples obtained were re-dissolved in HPLC grade methanol (100 μL) by vortex-mixing (30 s) and centrifuged (1,000×*g*, 10 min, 10 °C) to remove any remaining biological materials. Samples (100 μL) were then analysed by HPLC using a C_18_ μBondapak™ column eluted with propan-2-ol in water (29 % v/v) adjusted to pH 3.2 with orthophosphoric acid with a fluorimetric detector (excitation 480 nm, emission 560 nm). Data were collected and analysed using a PowerChrom integrator and software programme (PowerChrom v 2.0.7). For each experiment, a Dox standard curve was prepared in parallel.

### Quantitation of total HPMA copolymer–Dox and released free Dox in tumour samples

As HPMA copolymer–Dox does not extract directly into the organic solvent, free Dox present in the tumour samples could be extracted and quantified as described above [20, 21]. To quantify HPMA copolymer–Dox, a previously described acid hydrolysis method [20, 21] was used to first liberate the Dox aglycone from the conjugate as follows. Tumours were homogenised in PBS (2,000 μL). Then to duplicate samples (900 μL), Dnm was added as an internal standard (500 ng Dnm; 100 μL in PBS) followed by HCl (2 M, 1 mL) before heating at 80 °C for 20 min. Samples were neutralised by the addition of NaOH (2 M; 1 mL), ammonium formate buffer (1 M; pH 8.5; 1.5 mL) and then chloroform/propan-2-ol (4:1 v/v, 5 ml) were added, and the samples were extracted. HPLC analysis was conducted exactly as described above for free Dox. To ensure quantitation, HPMA copolymer–Dox standards were also analysed in parallel.

### Data expression

Data describing tumour accumulation of Evans Blue or HPMA copolymer–Dox were expressed as either (i) the  % administered dose per tumour (for the conjugate the value is given as % Dox-equiv dose administered) or (ii) the administered dose % dose/g tumour). Data shown are mean ± SE. Statistical significance was calculated using Student’s *t* test for comparison of the mean of two small samples. Statistical differences of at least *p* < 0.05 were considered statistically significant. To allow comparison between “small” and “large” tumours, the three smallest and three largest tumours available were grouped according to the classifiers for “small” and “large” described in the figure legends where tumour size as a variable is examined.

## Results

### Accumulation of Evans Blue, FCE28068 and free Dox in s.c. B16F10 and L1210 tumours

**Fig. 2:**
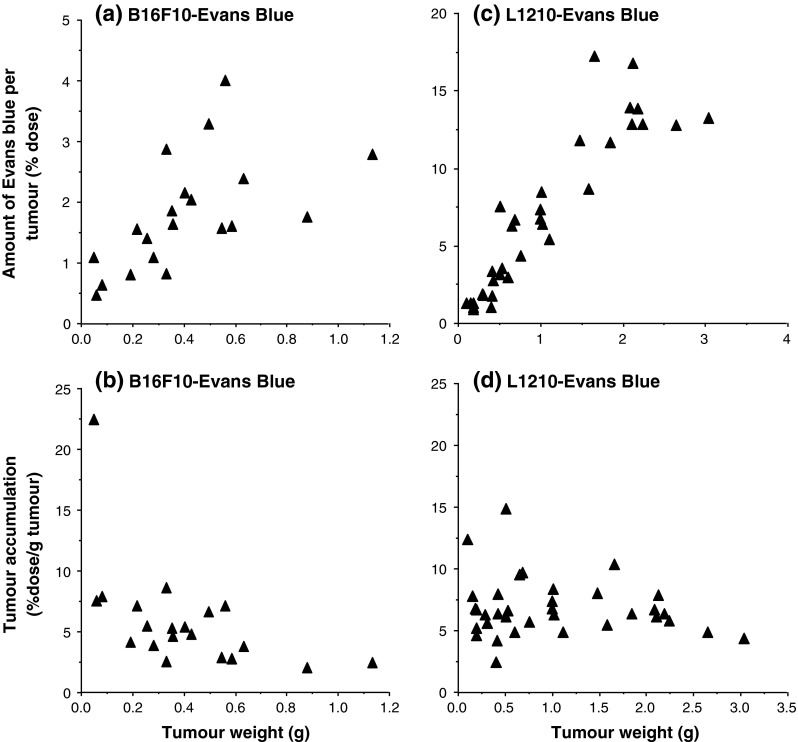
Levels of Evans Blue detected in s.c. B16F10 and L1210 tumours at 1 h after i.v. administration. **a**, **c** Tumour levels expressed as % dose per tumour and **b**, **d** tumour levels expressed as % dose/g/tumour. Each point represents a single tumour sample

**Fig. 3:**
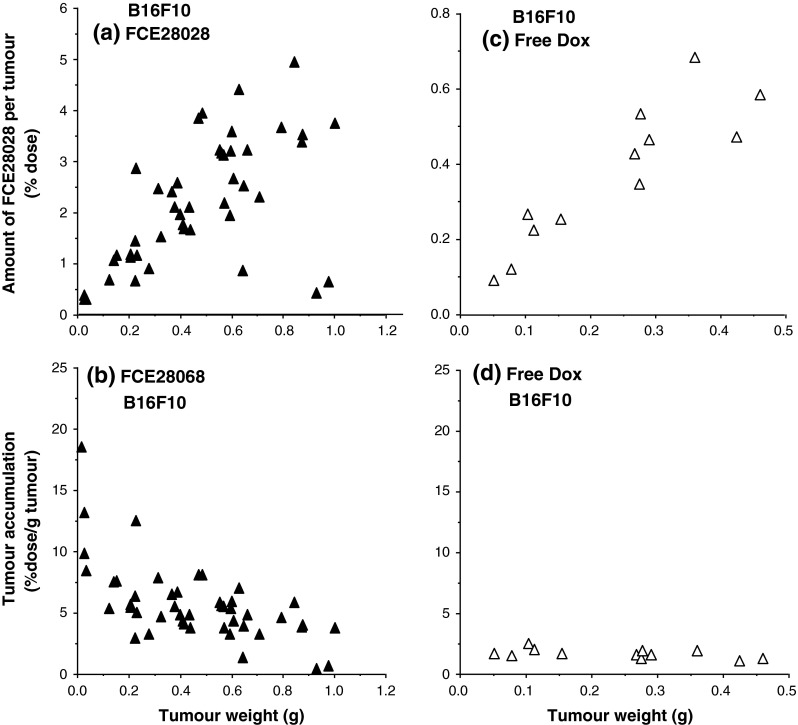
Levels of FCE28068 and free Dox detected in s.c. B16F10 tumours at 1 h after i.v. administration. **a**, **c** Tumour levels expressed as % dose per tumour, and **b**, **d** tumour levels expressed as % dose/g/tumour. Each point represents a single tumour

**Fig. 4:**
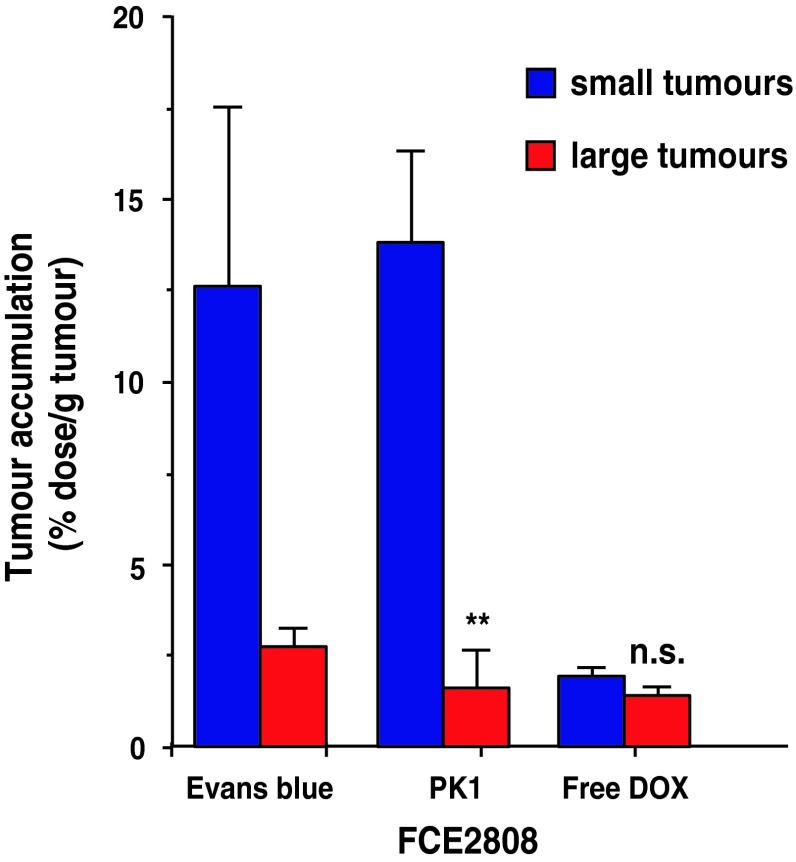
Comparison of the levels of FCE28068, Evans Blue and free Dox detected in small (<100 mg) and large (>400 mg) B16F10 tumours (mean ± SE). Statistical significance *p < 0.02; n.s. not significantly different

To assess evidence for the operation of the EPR effect, intratumour levels of Evans Blue, FCE28028 and Dox detected at 1 h following i.v. administration in mice bearing s.c. B16F10 or L1210 tumours are shown in Figs. 2 and 3. When the tumour levels were expressed as % dose for Evans Blue (Fig. 2a, c), tumour levels increased in an approximately linear fashion with increasing tumour size up to a maximum of 4–6 % dose/tumour for the B16F10 model and ~15 % dose/tumour for the L1210 model (Fig. 2a, c). Values measured for Evans Blue and FCE28028 (Fig. 3a) were quantitatively similar across the B16F10 tumour size range, as expected for agents requiring permeable blood vessels for distribution. In contrast, tumour Dox values measured following i.v. administration of free drug were consistently much lower (<0.8 % dose per tumour; Fig. 3c), and expression in terms of % dose/g tumour tissue showed Dox localisation to be constant across all tumour sizes studied (in the range 1.1–2.5 % dose/g tumour; Fig. 3d). In contrast, Evans Blue (Fig. 2b) and FCE28028 (Fig. 3b) levels expressed as % dose/g tumour decreased as tumour size increased (Figs. 2b, 3b). The highest levels of 7.5–18 % dose/g were recorded in the smallest tumours (≤100 mg). Values fell to 0.4–3.7 % dose/g in the largest tumours (>600 mg; Fig. 3b). The observed tumour size dependence of Evans Blue accumulation while still evident was less marked in the L1210 model (Fig. 2d). Figure 4 summarises data with respect to size of B16F10 tumours in relation to accumulated systemically administered Evans Blue, FCE28028 and Dox.

### Comparison of FCE28068 levels in murine and human xenograft tumours

**Fig. 5:**
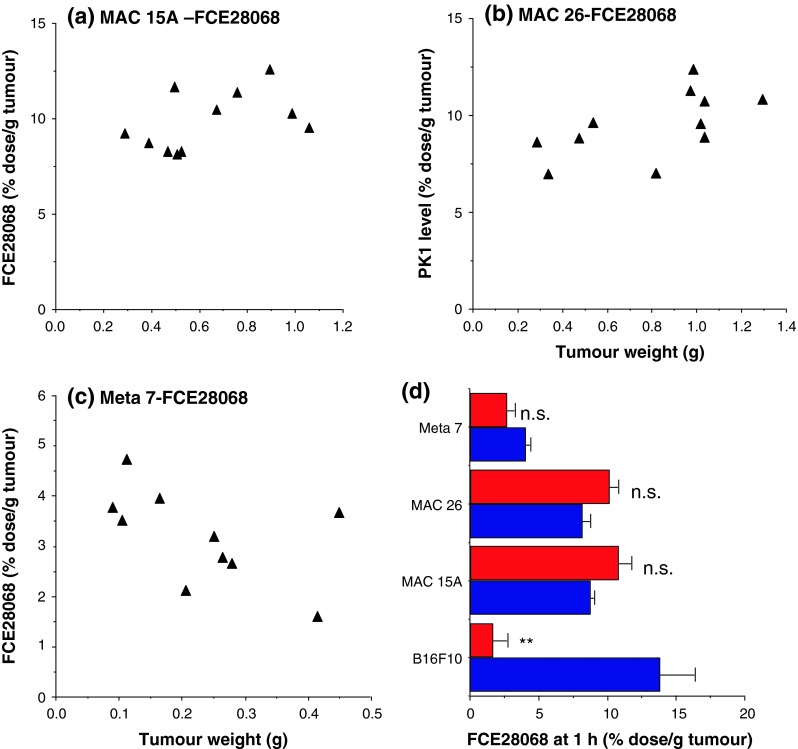
FCE28068 levels in MAC 15A, MAC 26 and Meta-7 tumours at 1 h after i.v. administration. The data in **a**–**c** individual tumour samples and **d** a comparison of the levels after measured in the three smallest and three largest tumours. The distribution of FCE 28608 for the B16F10 model is shown for comparison. In Fig. 5 “small” B16F10, MAC 15A MAC 26 and Meta-7 tumours were <100, <470, <480 and <150 mg, respectively. “Large” B16F10, MAC 15A, MAC 26 and Meta-7 tumours were >400, >900, >1,000 and >300 mg, respectively. Statistical significance ** *p* < 0.02; *ns* not significantly different

In contrast to the results obtained in the B16F10 models, when FCE28068 was administered to mice bearing MAC 15A (8.2–12.6 % dose/g), MAC 26 adenocarcinoma (6.9–10.8 % dose/g) or Meta-7 lung (3.5–4.7 % dose/g) tumours (Fig. 5), the amount of conjugate in the tumour did not show statistically significant changes as tumour size increased. While the MAC tumours accumulated FCE28068 to a similar degree as the B16F10 model, approximately two to three lower conjugate levels were seen in the Meta-7 lung model (Fig. 5d).

**Fig. 6:**
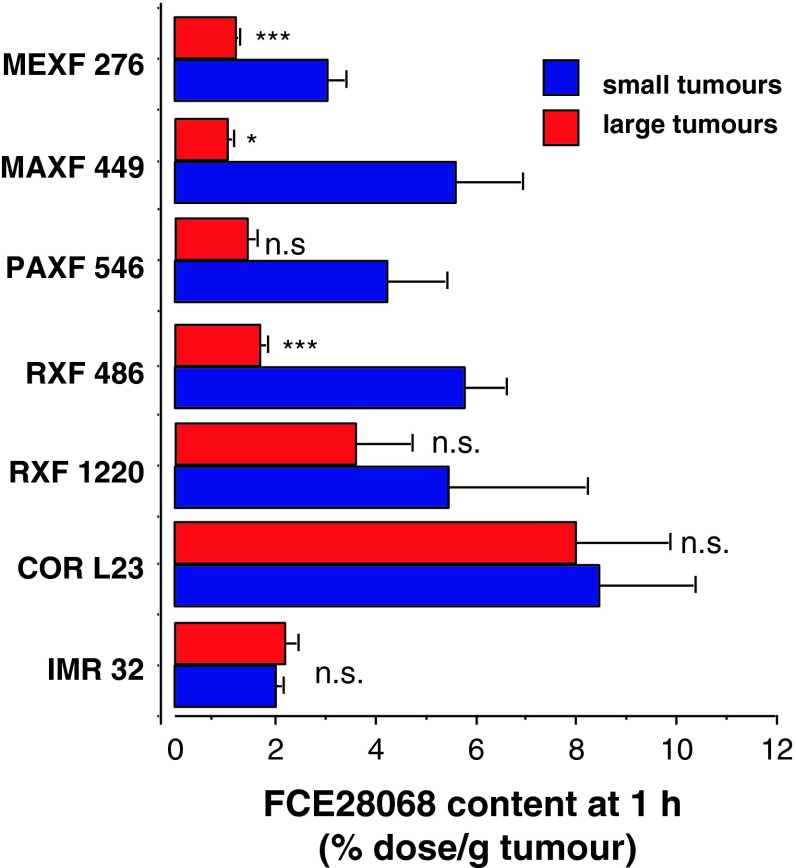
Comparison of the levels of FCE28068 detected in small and large human xenograft tumours at 1 h (mean ± SE, *n* = 3). Tumour model size cut-offs (mg small, mg large) are as follows: MEXF276 (<50, >200); MAXF449 (<50, >200), PAXF 546 (<100, >500), RXF 486 (<100, >600), RXF 1220 (<100, >400), CORL 23 (<100, >400), IMR32 (<120, >220). Statistical significance *p < 0.05 ***p < 0.01, *ns* not significantly different. For the full data set, see Supplementary Figs. 1 and 2

We therefore examined a series of additional models, as a tumour size-dependent nanomedicine delivery has been incompletely explored, and the existence of this phenomenon has important implications for the design of pre-clinical and clinical studies with nanomedicines. The MEXF 276, MAXF 449 and RXF 486 human xenograft models displayed a tumour size-dependent pattern of FCE28068 accumulation (supplemental Fig. 1). Although PAXF 546 showed a similar trend, it should be noted that the three smallest and the three largest tumours of PAXF 546 pancreatic carcinoma tumours did not give statistically significance different values. In contrast, the RXF 1220, COR L23, IMR 32, SK-N-SH and SK-N-DZ tumours (supplemental Fig. 2) displayed size-independent accumulation of FCE28068. (Only two SK-N-DZ tumours were available, but the data are included for completeness). Comparison of summary mean values obtained for FCE28068 accumulation in all the tumour xenografts (Fig. 6) revealed the highest values in the non-small cell lung cancer COR L23 (4.7–12.2 % dose/g) and the lowest values in the larger (0.2–0.4 g) MAXF 449 tumours (1.0 ± 0.1 % dose/g). Early-phase tumour levels of FCE28068 across all the xenograft tumours (and sizes) examined displayed ~12-fold variation in magnitude.

### Dox released from FCE28068 in murine and human xenograft tumours

**Fig. 7:**
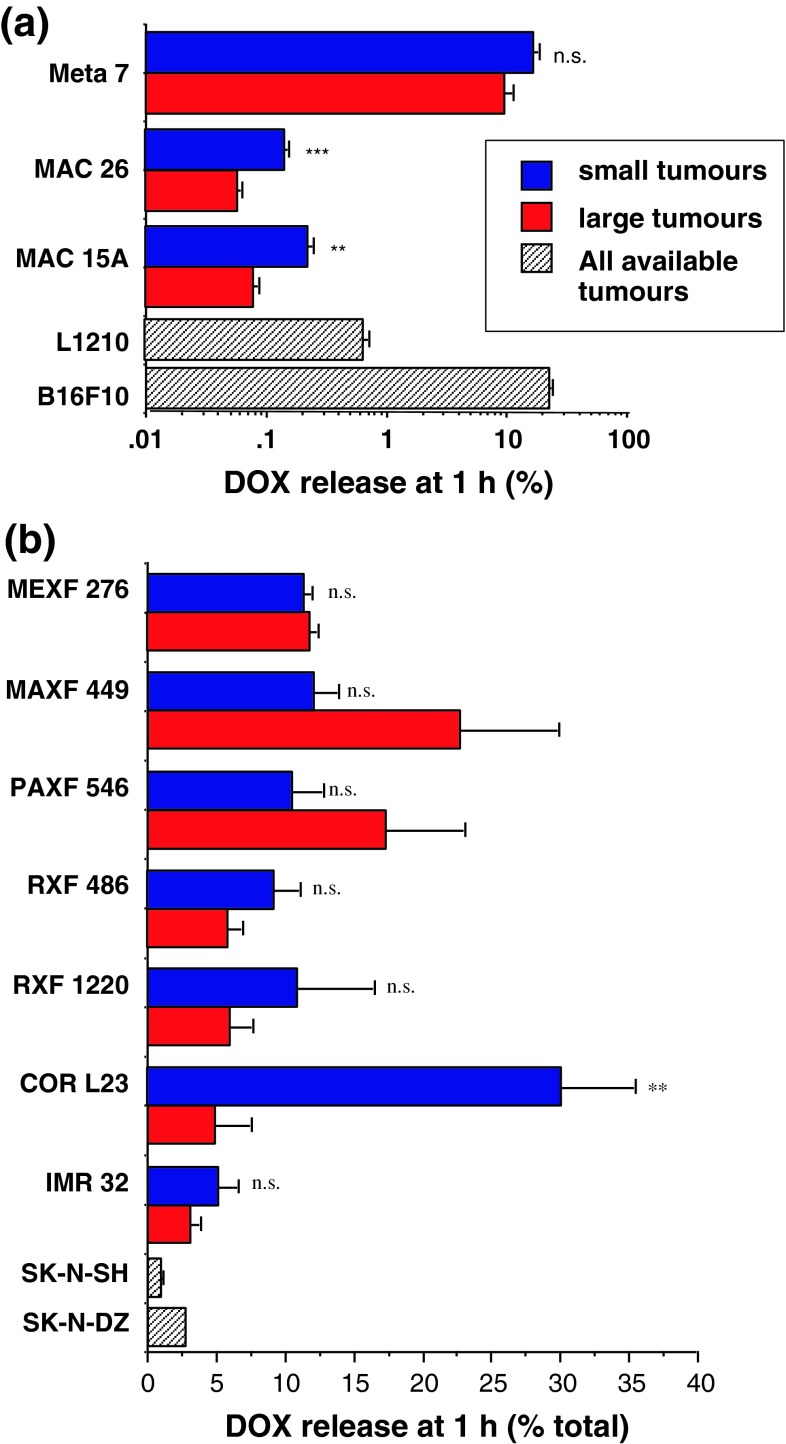
Comparison of the DOX released from FCE28068 in the different tumour models. *Panel* (**a**) murine tumour models (mean ± SE, *n* = 3). Statistical significance: **p < 0.02; ***p < 0.01; *ns* not significantly. *Panel* (**b**) human xenograft models (mean ± SE, *n* = 3; except SK-N-SH, *n* = 5 and SK-N-DZ, *n* = 2). Statistical significance: ** *p* < 0.02; *ns* not significantly different. Tumour size classification as per Figs. 4 and 6

After administration of FCE28068, the free DOX detected in all tumour samples at 1 h expressed as percentage of total Dox (i.e. conjugated drug + free) is shown in Fig. 7. Only the MAC 15A, MAC 26 murine tumours and the COR L23 xenograft showed tumour size dependency in terms of Dox liberation at 1 h. This is interesting as none of these tumour models displayed tumour size-dependent FCE28208 levels at 1 h. Dox release was greater in the smaller tumours, and the difference is particularly striking for the smaller (<100 mg) COR L23 tumours. Notably, the extent of Dox release in both MAC tumours was very low (supplemental Fig. 3). Overall, there were a >200-fold difference in the free Dox levels seen at 1 h in the murine models studied and a 30-fold variation in the xenograft models. The fastest Dox release was observed in B16F10 (22.9 ± 1.2 % at 1 h) and the smaller in COR L23 tumours (30.1 ± 5.3 %) with the slowest release rate observed in the larger MAC 26 tumours (0.06 ± 0.01 %) and the SK-N-SH neuroblastoma (1.0 ± 0.1 %).

## Discussion

In vitro and in vivo pre-clinical models used to screen novel low molecular weight anticancer agents and biologics have evolved significantly to better mimic the clinical disease setting (reviewed in [22, 23]). While these advances give hope of lead compounds with an increased probability of a successful clinical outcome, many of the in vitro and in vivo methods/models used today are not optimal for nanomedicine evaluation given their very different cellular and whole-body pharmacokinetics compared to low molecular weight agents (discussed in [2]). Following i.v. administration, low molecular weight drugs distribute rapidly throughout the body with little or no tumour selectivity (evidenced here for Dox in Fig. 3d). After this distribution phase, typically <0.1 % of the administered drug dose is recoverable in the circulation, usually in the form of metabolites and/or protein-bound drug. In contrast, nanomedicines (including liposomes, polymer conjugates and nanoparticles) are retained within the bloodstream due to the tight vascular endothelial barrier present in most organs and limitation of cellular uptake to the endocytic pathways (discussed in [24]). In the absence of receptor-mediated targeting, nanomedicine blood clearance is largely governed by the rate of reticuloendothelial system (RES) uptake and/or renal elimination. Arriving blood concentration is the primary driver for passive tumour targeting (Fig. 1b), and a low EPR effect is typically seen when a nanocarrier is cleared rapidly by professional phagocytes. However, it widely recognised that thereafter vasculature complexity (e.g. different classes of angiogenic vessels [25], vessel disorganisation and heterogeneity of tumour perfusion) and intratumoural biological barriers (e.g. high interstitial pressure, the extracellular matrix, coupled with the presence or absence of lymphatic drainage, etc. [2, 10, 26]) play an important role in passive targeting ultimately achieved in any particular tumour mass.

In the 1950s and 1960s, radio-iodinated albumin and albumin aggregates were used clinically to image tumours. Unknowingly, these imaging agents were already capitalising on the EPR effect for tumour selectivity [27], as do all chemotherapeutic and phototherapy agents that bind to plasma proteins. Using an Evans Blue–albumin complex, Matsumura and Maeda [5] visualised and quantified tumour-specific passive accumulation in a Sarcoma 180 model (32 % dose/g tumour at 48 h). In the experiments reported here, Evans Blue and FCE28068 accumulation by L1210 and B1F10 (Figs. 2, 3) tumours was in the range ~1–22 % dose/g at 1 h. There was good correlation between Evans Blue and the nanomedicine FCE 28068 across the tumour size range. The data are in agreement with the previous reports describing Evans Blue [17], HPMA copolymer fractions (molecular weight from 4.5 to 800 kDa), PAMAM dendrimer-Dox and liposomal doxorubicin tumour accumulation. Rapid passive targeting gave similar tumour values (% dose/g) irrespective of polymer molecular weight or construct architecture [28–31].

A significant observation in these experiments is a 12-fold variation in functional EPR, with many models displaying tumour size-dependent accumulation. The higher tumour localisation was noticeably in the smaller tumours, and this maybe a reflection of greater angiogenic activity in smaller tumours, reduced or absent tumour blood supply in hypoxic regions of larger necrotic tumours, an increase in tumour interstitial fluid pressure as the tumour grows, or a combination of all of these factors. ^111^In-DTPA-labelled PEGylated liposomes injected i.v. to mice bearing KB human head and neck squamous cell xenografts also displayed tumour levels inversely correlated with tumour size: 15.1 ± 10.8 % dose/g in tumours of <0.1 g and 3.0 ± 1.3 % dose/g in tumours of >1 g [31]. In this regard, the current results of experiments raise the possibility of a novel development strategy for nanomedicines, focusing on systemic delivery of such constructs in the adjuvant setting or with “small-volume” metastatic disease after surgical debulking.

Whatever the mechanism, there is a growing body of evidence that nanosized vectors show greatest targeting to the smaller tumours and this could provide an important opportunity to localise to those micrometastases so difficult to diagnose and treat effectively. Use of ^111^In-DTPA-labelled PEGylated liposomes to image 17 patients with locally advanced cancers [13] gave positive tumour images in 15/17 patients and tumour levels in the range of ~0.5–3.5 % dose at 72 h. Highest liposome localisation was observed in patients with head and neck cancers (~0.033 % injected dose/g tumour) (also noted for FCE2068 in Phase I clinical studies [11]) and lower levels in breast cancers (~0.005 % injected dose/g). The breast tumours were larger than the lung tumours, supporting a relationship between small tumour site and improved localisation, but the relationship was not unequivocal as it was noted that some tumours of similar size but different histological type displayed different levels of liposomal uptake. Using ^99m^Tc-DTPA-labelled PEGylated liposomes that localised to lung and head and neck cancers [32], others have suggested a correlation between tumour targeting and microvessel density as defined by anti-CD31 staining. Although tumour vessel density (VEGF expression) does correlate with tumour vascular permeability (Evans Blue) in some human tumour xenografts [17], other studies have indicated that a more complex multifactorial mechanism underpins EPR-mediated targeting and efficacy, e.g. inflammatory status. At this time, it is difficult to predict tumour localisation/antitumour activity using a single biomarker [14].

Overexpression of lysosomal thiol-dependent proteases in many human tumours has been well documented [33], and these enzymes play an important role in tumour progression (e.g. metastasis and angiogenesis). A growing number of polymer conjugates are being designed for lysosomotropic delivery [34, 35] and cathepsin B-mediated drug release [1, 2]. The wide variation in early-phase Dox release from FC28068 (~200-fold) observed here illustrates the need to characterise tumour models around the basis for delivery to tumours and response of tumours to nanomedicines as part of evolving an optimal clinical strategy for their development. For example, retrospective analysis of clinical data from PPX Phase III trials in advanced lung cancer patients [36] showed improved survival in female but not in male patients, and it has been postulated that patient oestradiol level might play a pivotal role as oestrogens are known to induce cathepsin B activity. Earlier studies involving FCE28068 in s.c. B16F10 tumours [15, 37] indicated an ~30 min time lag before Dox release began, supporting the hypothesis of drug release following endocytic internalisation [38]. However, it should be noted that other factors may play a role in controlling the rate of drug release: (i) rate of conjugate diffusion through the tumour interstitium [39], (ii) the rate of endocytic internalisation (endocytic gateways and intracellular trafficking pathways are often dysregulated in cancer [40]) and (iii) exposure to cathepsin B in the extracellular milieu. This complexity underlines the importance of functional tumour calibration in terms of drug release and also the future role of the real-time tumour imaging techniques that are emerging for quantitation of proteolysis per se [41] and polymer degradation [42].

## Conclusions

The wide variation in passive targeting (% dose/g) and Dox liberation observed here (1 h) highlights the need to calibrate in vivo tumour models in respect of these parameters before they are used to define pharmacokinetics and/or antitumour activity of nanomedicines. This would provide more clinically relevant models for optimisation of lead candidates and benchmarking performance against anticancer nanomedicines already in routine clinical use [2]. Evans Blue is a useful tool for routine evaluation of passive targeting, and the 1 h time point enables comparison of different nanocarriers minimising complications arising due to different blood clearance rate, tumour efflux and intratumoural degradation of the probe over time. These observations also emphasise the potential to select patients for early clinical trial involving nanomedicine therapy that are most likely to respond, i.e. by use of clinical imaging to verify functional EPR [43], and monitoring of tumour biopsy samples for biomarkers relevant to activating conditions, e.g. in this case cathepsin B.

## Electronic supplementary material

Below is the link to the electronic supplementary material.

Fig. 1 FCE28068 levels in MEXF 276, PAXF 546, MAXF 449 and RXF 486 tumours at 1 h after i.v. administration. The data in panels (a) - (d) show individual tumours. (PPT 165 kb)

Fig. 2 FCE28068 levels in RXF 1220, IMR 32, COR L23, SK-N-SH and SK-N-DZ tumours at 1 h after i.v. administration. The data in panels (a) - (e) show individual tumours. (PPT 178 kb)

Fig. 3 Summary of tumour uptake of FCE28068 and the rate of DOX release in the MAC tumours (mean ± SE, n = 11). **(PPT 146** **kb)**
