# Supplementary material for: Validation of tumour models for use in anticancer nanomedicine evaluation: the EPR effect and cathepsin B-mediated drug release rate
Source: Cancer Chemother Pharmacol. 2013 Jun 25;72(2):417–27. doi: 10.1007/s00280-013-2209-7 (PMC3718995; doi:10.1007/s00280-013-2209-7)
Supplement: Supplementary file 1 — Fig. 1 FCE28068 levels in MEXF 276, PAXF 546, MAXF 449 and RXF 486 tumours at 1 h after i.v. administration. The data in panels (a) - (d) show individual tumours. (PPT 165 kb) [file 280_2013_2209_MOESM1_ESM.ppt]

## Slide 1
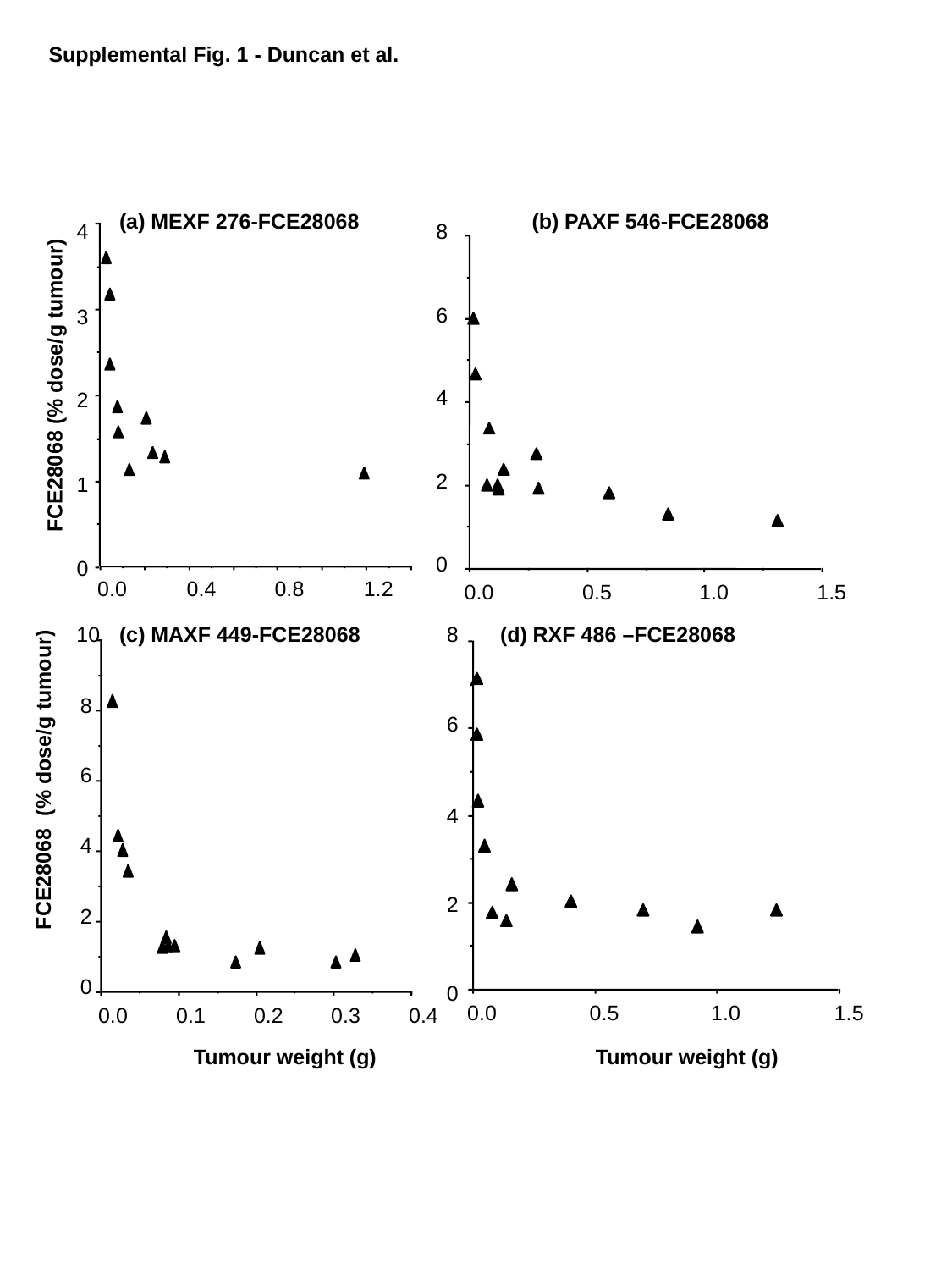

Supplemental Fig. 1 - Duncan et al.
(a) MEXF 276-FCE28068
(b) PAXF 546-FCE28068
8
6
4
2
0
0.0
0.5
1.0
1.5
4
3
FCE28068 (% dose/g tumour)
2
1
0
0.0
0.4
0.8
1.2
10
8
6
4
2
0
(c) MAXF 449-FCE28068
8
6
4
2
0
(d) RXF 486 –FCE28068
FCE28068 (% dose/g tumour)
0.0
0.5
1.0
1.5
0.0
0.1
0.2
0.3
0.4
Tumour weight (g)
Tumour weight (g)
