# Supplementary material for: Validation of tumour models for use in anticancer nanomedicine evaluation: the EPR effect and cathepsin B-mediated drug release rate
Source: Cancer Chemother Pharmacol. 2013 Jun 25;72(2):417–27. doi: 10.1007/s00280-013-2209-7 (PMC3718995; doi:10.1007/s00280-013-2209-7)
Supplement: Supplementary file 2 — Fig. 2 FCE28068 levels in RXF 1220, IMR 32, COR L23, SK-N-SH and SK-N-DZ tumours at 1 h after i.v. administration. The data in panels (a) - (e) show individual tumours. (PPT 178 kb) [file 280_2013_2209_MOESM2_ESM.ppt]

## Slide 1
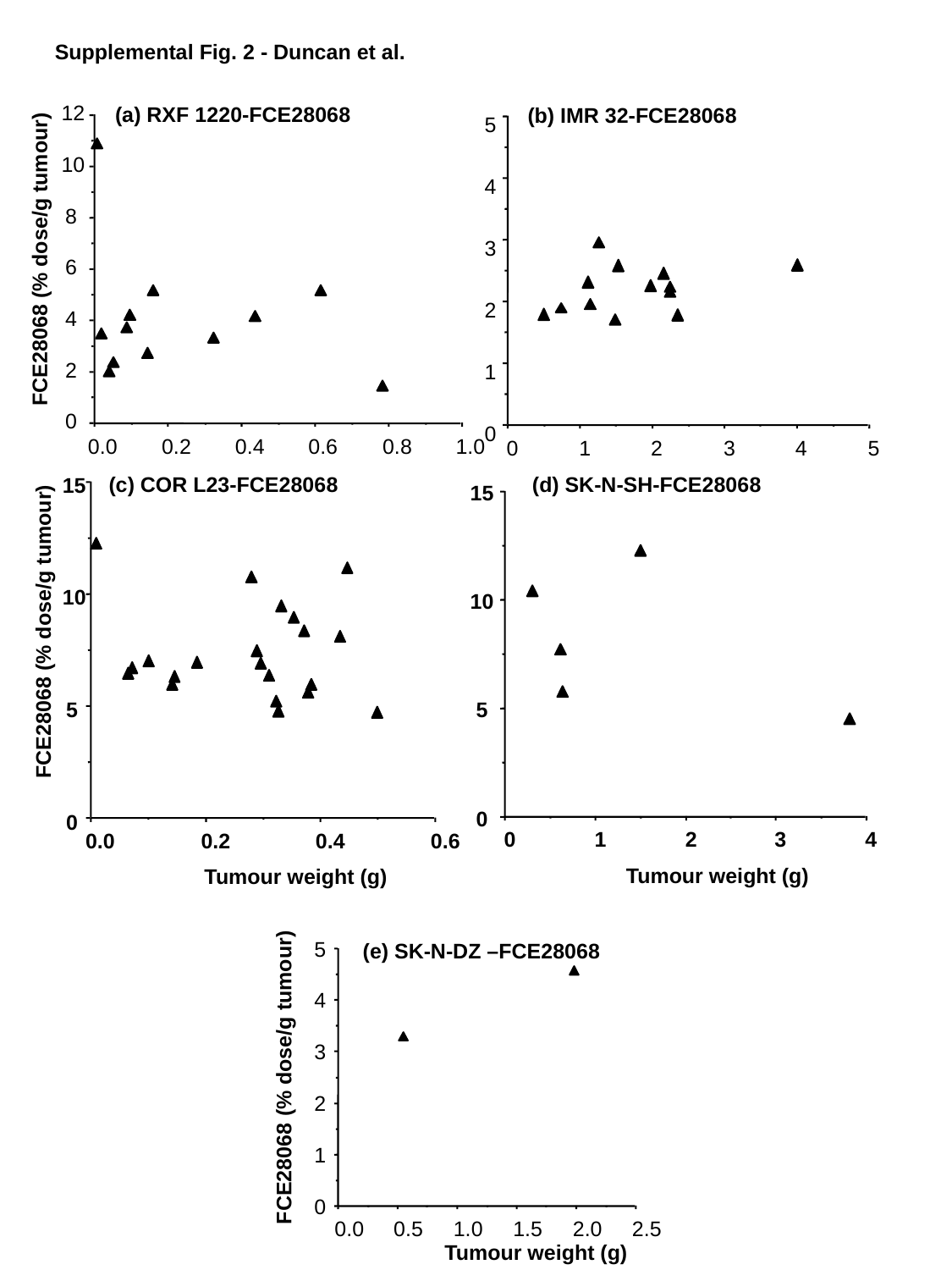

Supplemental Fig. 2 - Duncan et al.
12
10
8
6
4
2
0
FCE28068 (% dose/g tumour)
0.0
0.2
0.4
0.6
0.8
1.0
(a) RXF 1220-FCE28068
(b) IMR 32-FCE28068
5
4
3
2
1
0
0
1
2
3
4
5
(c) COR L23-FCE28068
(d) SK-N-SH-FCE28068
15
10
5
0
15
10
5
0
FCE28068 (% dose/g tumour)
1
2
3
0
4
0.0
0.2
0.4
0.6
Tumour weight (g)
Tumour weight (g)
5
4
3
2
1
0
(e) SK-N-DZ –FCE28068
FCE28068 (% dose/g tumour)
0.0
0.5
1.0
1.5
2.0
2.5
Tumour weight (g)
