# Supplementary material for: Validation of tumour models for use in anticancer nanomedicine evaluation: the EPR effect and cathepsin B-mediated drug release rate
Source: Cancer Chemother Pharmacol. 2013 Jun 25;72(2):417–27. doi: 10.1007/s00280-013-2209-7 (PMC3718995; doi:10.1007/s00280-013-2209-7)
Supplement: Supplementary file 3 — Fig. 3 Summary of tumour uptake of FCE28068 and the rate of DOX release in the MAC tumours (mean ± SE, n = 11). (PPT 146 kb) [file 280_2013_2209_MOESM3_ESM.ppt]

## Slide 1
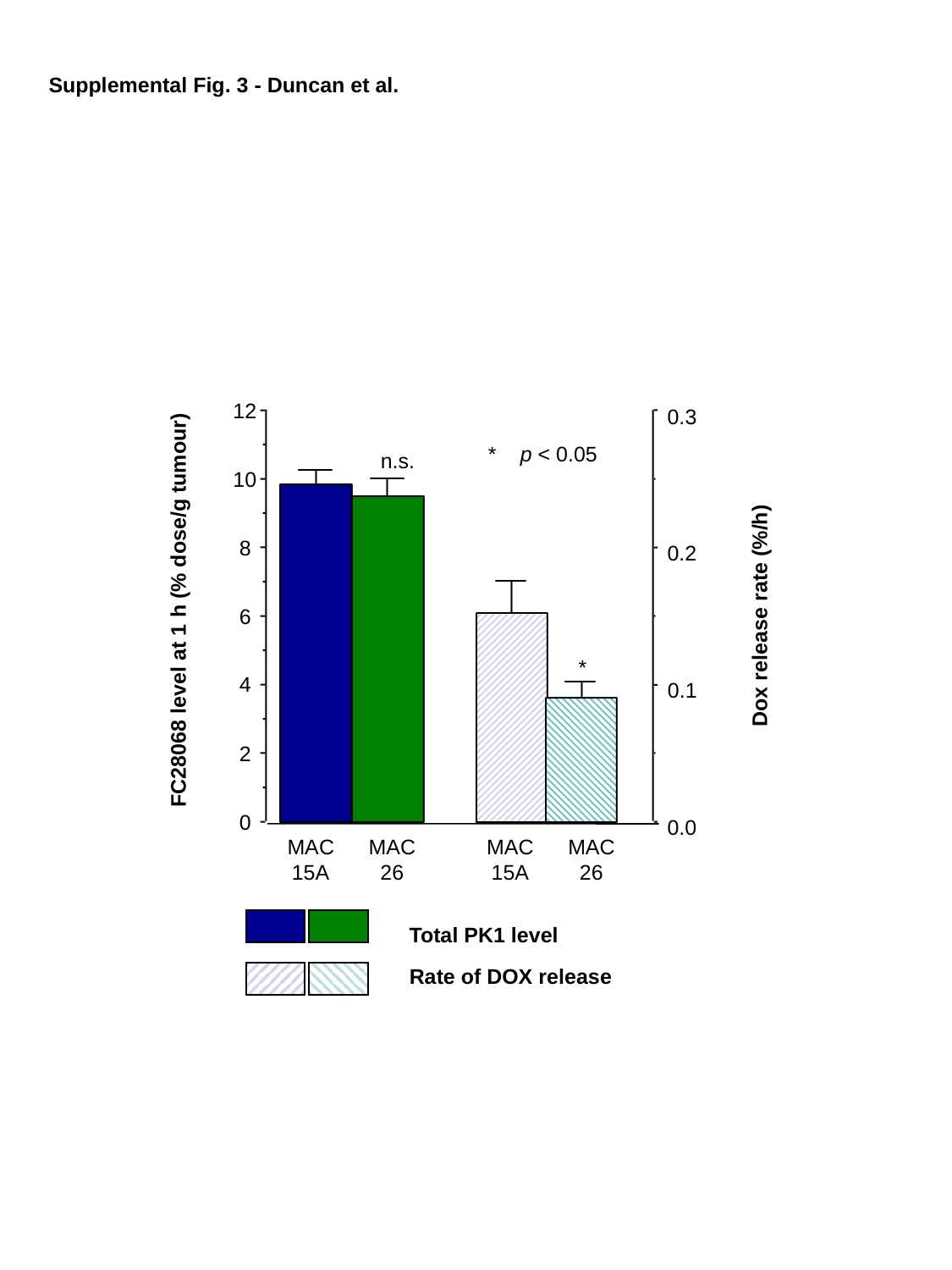

Supplemental Fig. 3 - Duncan et al.
12
0.3
0.2
0.1
0.0
* p < 0.05
n.s.
10
8
FC28068 level at 1 h (% dose/g tumour)
Dox release rate (%/h)
6
*
4
2
0
MAC
15A
MAC
26
MAC
15A
MAC
26
Total PK1 level
Rate of DOX release
